# Synchronized diaphragmatic stimulation for the treatment of HFrEF—a review

**DOI:** 10.1007/s10741-025-10525-y

**Published:** 2025-05-28

**Authors:** L.R. Goldberg, M. Mirro, G. Becker, T. Shaburishvili, M. Fudim

**Affiliations:** 1 University of Pennsylvania Perelman School of Medicine, Philadelphia, PA USA; 2 Indiana University School of Medicine and Parkview Mirro Center for Innovation, Fort Wayne, IN USA; 3 University of George and Tbilisi Heart and Vascular Clinic, Tbilisi, Georgia; 4 Duke Clinical Research Institute and Duke Cardiology, Durham, NC USA; 5 Ilia State University and Tbilisi Heart and Vascular Clinic, Tbilisi, Georgia

**Keywords:** Heart failure, HFrEF, Synchronized diaphragmatic stimulation, GDMT, Intrathoracic pressure, Minimally invasive

## Abstract

The gap between maximally tolerated medical therapy and consideration for permanent mechanical circulatory support and/or cardiac transplant or palliative treatment of moderate to severe heart failure represents an underserved patient population. New therapies are evolving which may not only improve quality of life for these patients but also improve hemodynamics and potentially reverse the progression of the disease. This review is focused on one such therapy, synchronized diaphragmatic stimulation. Current clinical results suggest that patients experience improved exercise tolerance, quality of life, and hemodynamic function over 6–12 months of therapy which can be safely implemented through a minimally invasive laparoscopic procedure, often as an outpatient. This technology has been granted breakthrough device designation and is being evaluated for a double-blinded, randomized controlled trial by the US FDA.

## Background

Heart failure is a syndrome with progressive symptomatology resultant from several etiologies including ischemia, hypertension, valvular disease, viral infections, etc. The majority of patients with heart failure and reduced left ventricular ejection fraction (HFrEF) continue to experience symptoms even after their guideline-directed medical therapy (GDMT) is optimized [1], quality of life is usually compromised, and instances of decompensation and rehospitalization are common with a reduced life expectancy [2]. Those HFrEF patients who are in sinus rhythm and have a prolonged QRS duration may be eligible for cardiac resynchronization therapy (CRT) as effective add-on therapy; however, there are twice as many HFrEF patients who are not eligible for CRT and whose need is unmet [3]. These patients comprise the population at the transition from stage C to stage D heart failure [1] and may benefit from a layered approach of additional medical and device therapies in addition to first-line GDMT to change the trajectory towards advanced therapies or palliation.

### Synchronized diaphragmatic stimulation (SDS)

The FDA provides breakthrough device designations for promising technologies to address diseases and conditions with unmet needs. Synchronized diaphragmatic stimulation (SDS) is a novel approach designed to improve cardiac function, symptoms and, ultimately, outcomes for heart failure patients who remain symptomatic while on GDMT. Recognizing the potential of this technology to impact those with moderate to severe heart failure, the FDA granted breakthrough device designation to expedite its clinical evaluation.

Synchronized diaphragmatic stimulation is a device based HFrEF therapy employing a dedicated, minimally invasive, laparoscopically implantable system. The device delivers stimulation pulses to the diaphragmatic muscle tissue (not to the phrenic nerve) causing localized contractions of the diaphragm that are imperceptible to the patient and coincident with the cardiac cycle to modulate intrathoracic pressure (ITP) and pericardial restraint. This stimulation improves cardiac performance through the recruitment of the diaphragm as a supplemental vascular pump [4, 5]. The SDS system consists of a subcutaneously implanted pulse generator (IPG), two leads that are affixed to the inferior diaphragm, and an external programmer to wirelessly adjust and monitor system functions, as well as on-board physiologic diagnostics.

### Pathophysiology

Elevated intracardiac pressures in relation to contractile power of the ventricles are an important pathological driver of heart failure progression and reduced cardiovascular performance. The degree of cardiac pressure elevation is determined by preload, afterload, and the pericardial restraint. Contractile power is defined by structural changes in the myocardium and the Frank-Starling law. The degree to which the pericardium restrains the heart is controlled by the pericardial structure and the intrathoracic pressure. The recognition of the role of both pericardial restraint and its relationship to ITP in heart failure is one of the fundamental concepts from which the SDS therapy is being developed [4, 5].

**Fig. 1:**
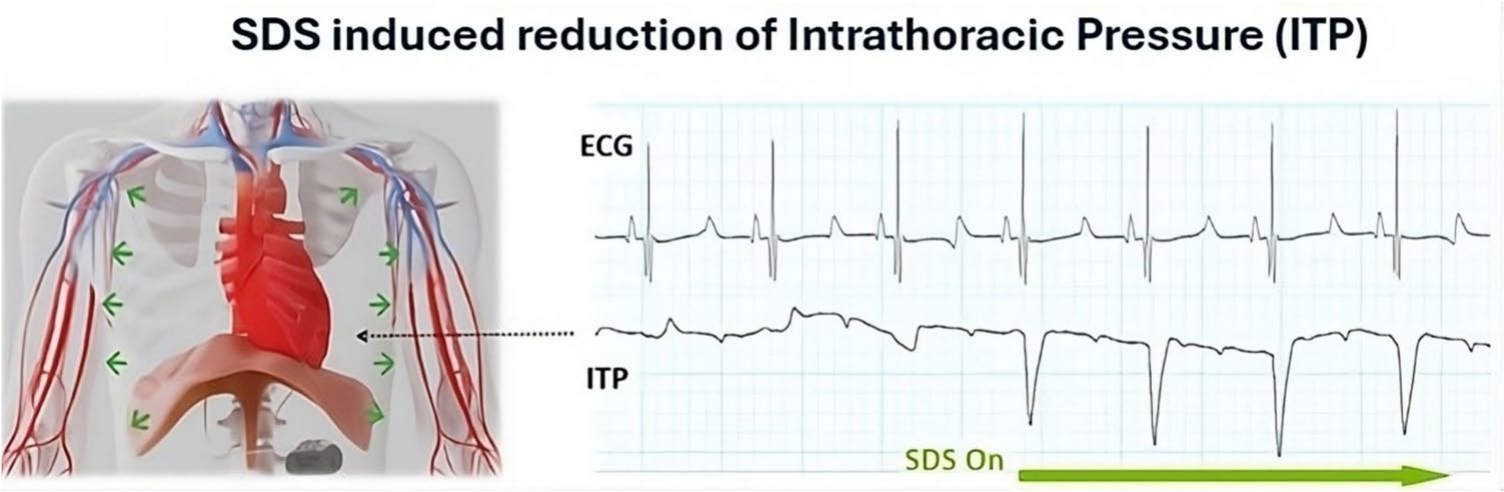
Illustration of intrathoracic pressure with SDS OFF and ON

The diaphragm is the major respiratory muscle responsible for inspiration and expiration; however, it also plays a physiological role during other voluntary and involuntary events such as coughing, sneezing and hiccups and non-respiratory events such as vocalization, swallowing, etc. It functions as a skeletal muscle during abdominal straining and as the thoracoabdominal pump that aids in the blood return to the heart during the normal respiratory cycle by decreasing ITP and increasing intraabdominal pressure (Fig. 1). The diaphragm also plays an underrecognized role in cardiac function and hemodynamics as manifested through phenomenon such as respiratory variations in heart rate on ECG and the use of voluntary coughing to clear the coronaries of contrast dye during transient bradycardias during coronary procedures.

Synchronized diaphragmatic stimulation coordinated with the cardiac cycle appears to successfully modulate ITP thereby impacting pericardial restraint and enhancing cardiac pre- and afterload.

## Implantation

Implantation of the SDS system combines the techniques of a routine two-port laparoscopy and the creation of a subcutaneous pocket typical of a cardiac implantable electrical device (CIED). The endoscopic surgeon inserts two trocars—one for the camera and the other to pass the leads. Under direct visual guidance the leads are attached using a custom laparoscopic tool designed to grasp the hub of the standard epicardial lead. The first lead is inserted immediately adjacent to the cardiac apex on the left inferior hemisphere of the diaphragm and the other on the right inferior hemisphere ensuring a critical mass of the heart lies in line with the electrical vector between the two leads.

**Fig. 2:**
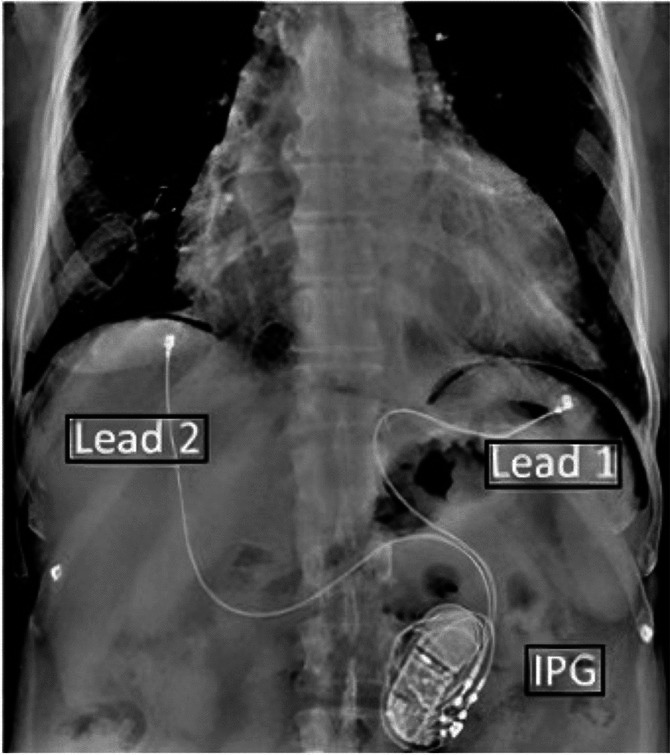
Position of implanted SDS system

The left lead is tested for bipolar sensing and stimulation of a small area of diaphragm near the cardiac apex to determine the capture threshold of the twitch. Next, the sensing performance is evaluated between the two leads to ensure adequate detection of the R-wave to which the diaphragmatic stimulus will be synchronized. Once adequate lead performance is achieved, a subcutaneous pocket is created adjacent to the port used to guide the leads. The terminal ends of the leads are tunneled to the pocket and connected to the pulse generator as they would be for a pacemaker. The two small trocar incisions are closed. The X-ray in Fig. 2 illustrates a typical SDS system following implant.

**Fig. 3:**
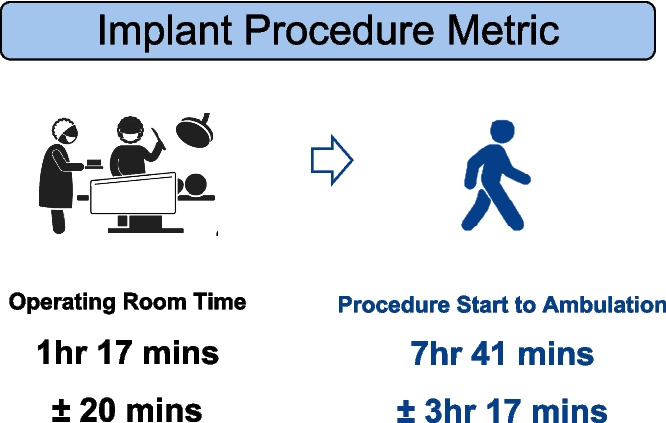
Procedure and recovery times from operating room start [8]

In the RECOVER-HF Pilot [6, 7] both implant procedure times and the time to ambulation demonstrate the safety and feasibility of providing SDS therapy in the outpatient setting (Fig. 3). After a short learning curve, the implantation time is 77 min from skin to skin and less than 8 h recovery time to ambulation.

Upon recovery from anesthesia, the SDS system is evaluated for sensing and capture prior to discharge and programmed for reliable R-wave detection and stimulation output to ensure capture without patient perception of the diaphragmatic twitch.

Importantly, there have been no device or procedure related adverse events in the human clinical trials to date [8]. The minimally invasive nature of the surgery provides the opportunity for these fragile patients to be treated quickly with short procedure times and minimal anesthesia time. This allows discharge the same or following day, reducing cost and minimizing the risks of infections or complications in this somewhat frail population. Another key benefit of the system is that the laparoscopic extra-vascular approach eliminates direct interfacing with the heart or vasculature preserving venous and endocardial space for a potential future CEID.

## Target population

Synchronized diaphragmatic stimulation is being implanted in HFrEF patients with NYHA class II/III, LVEF < 40%, narrow QRS (< 130 ms) with ongoing heart failure symptoms despite maximally tolerated guideline-directed medical therapy (GDMT). These patients are not candidates for CRT devices but have a progressive reduction in quality of life due to persistent or recurring symptoms despite optimized medical therapy. These patients are those who may transition from stage C to stage D heart failure facing consideration for advanced HF therapies such as LVAD or heart transplant or palliative care.

## Clinical results

The results from early feasibility trials have demonstrated clinically significant improvements in quality of life (QOL), exercise capacity, and cardiac performance. There is also emerging evidence of reverse ventricular remodeling as indicated by reduced end-systolic volumes at 1 year follow-up. During the 3-site, open-label first-in-human (FIH) trial of 15 patients, a threshold dose of 80% diaphragmatic synchronized stimulation to heart rate was necessary to achieve clinical benefit for all measured parameters [6]. This suggests that there is a dose effect of synchronized diaphragmatic stimulation similar to what has been observed in CRT pacing [9].

**Fig. 4:**
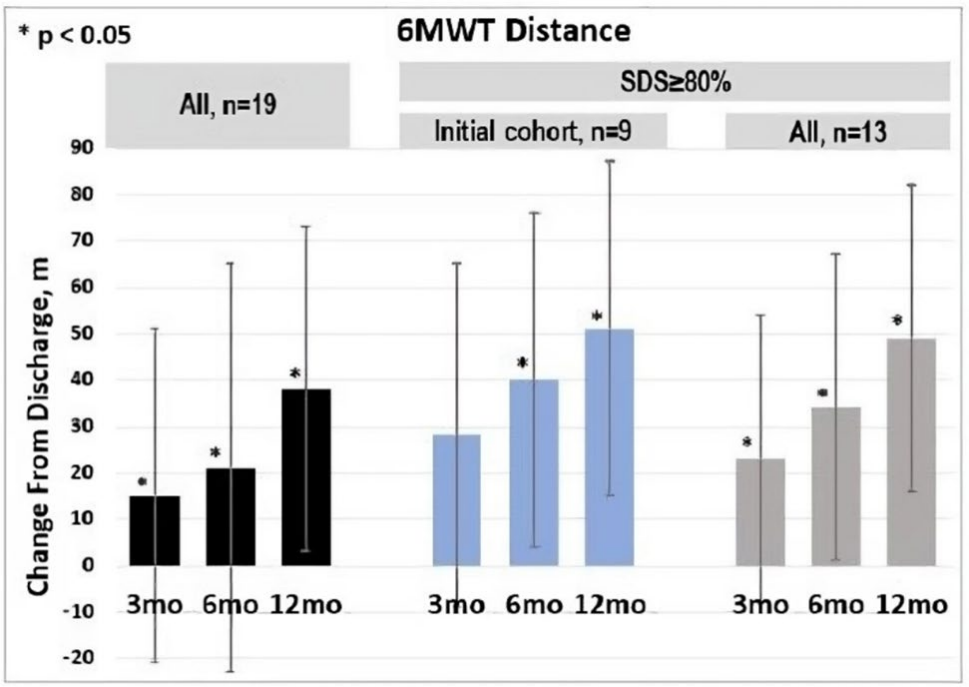
Improved exercise tolerance with early feasibility study (data on file at VisCardia, Inc.)

In data pooled from the first-in-human study (*N* = 15) and four additional patients treated to evaluate alternative implant techniques with the same device and follow-up, overall exercise capacity was improved by approximately 40 m on a 6-min hall walk test at 1-year post discharge in the full cohort and 50 m in those patients with greater than 80% synchronization (Fig. 4).

**Fig. 5:**
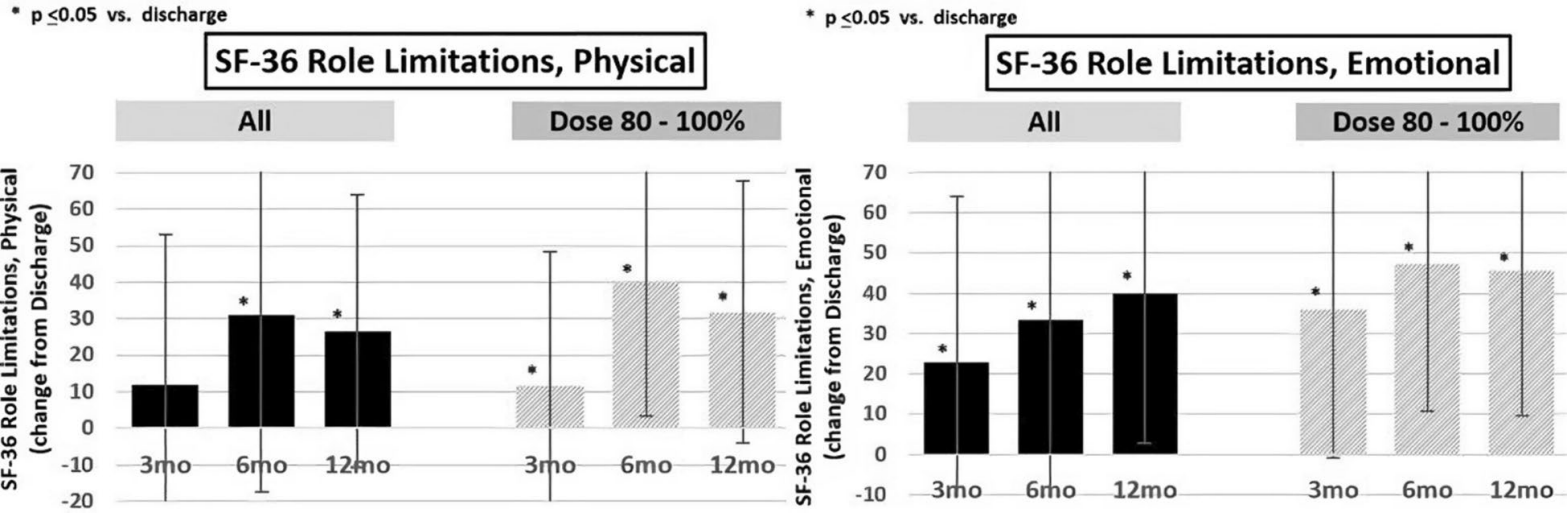
QOL improvement based on synchronized diaphragmatic pacing dose. First-in-human study (*N* = 15)

Figure 5 shows that although the entire cohort improved in QOL scores, those with greater than 80% successful synchronization (dose) showed greater improvement. The improvement is seen in both the physical and emotional components of the Short Form (SF-36) Health Survey.

**Fig. 6:**
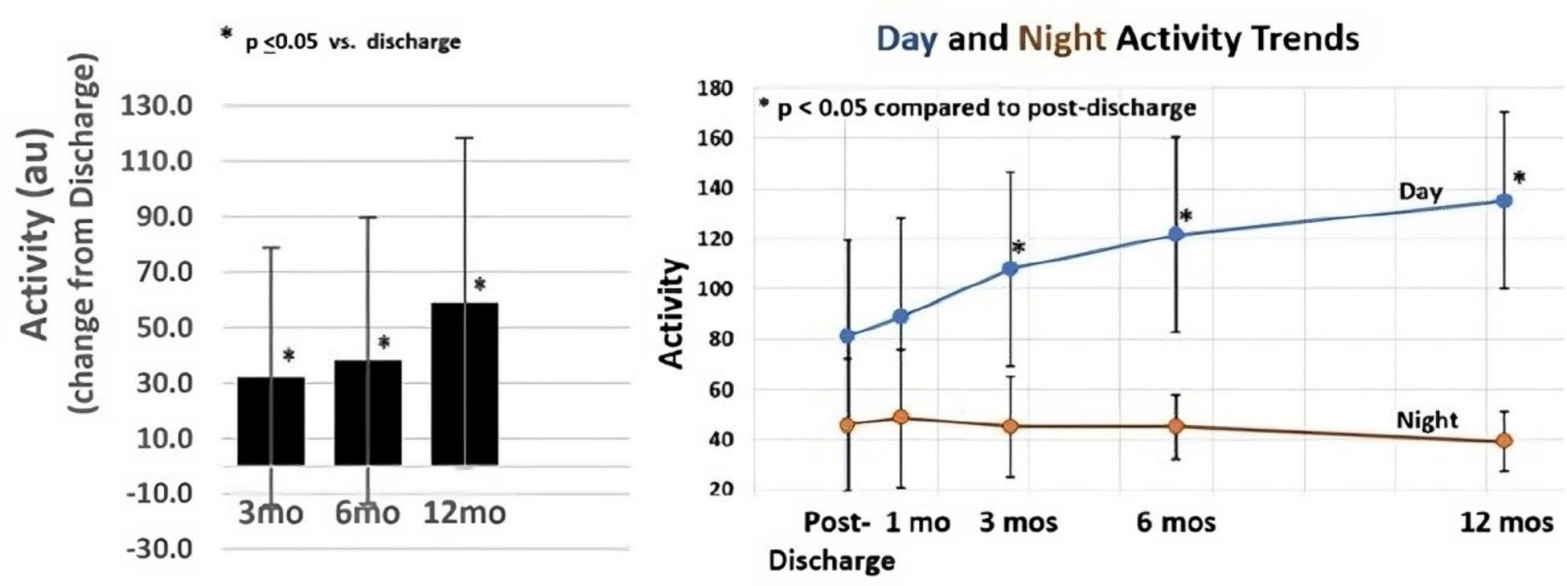
Physical activity as measured by an on-board diagnostic monitor. Total *N* = 19. Data on file at VisCardia, Inc

QOL is further illustrated in data pooled from the first-in-human study (*N* = 15) and four additional patients treated to evaluate alternative implant techniques with the same device and follow-up. The improvement in physical activity and restful sleep was measured by an on-board activity monitor showing progressive increased activity during the day and reduced activity while sleeping at night (Fig. 6).

**Fig. 7:**
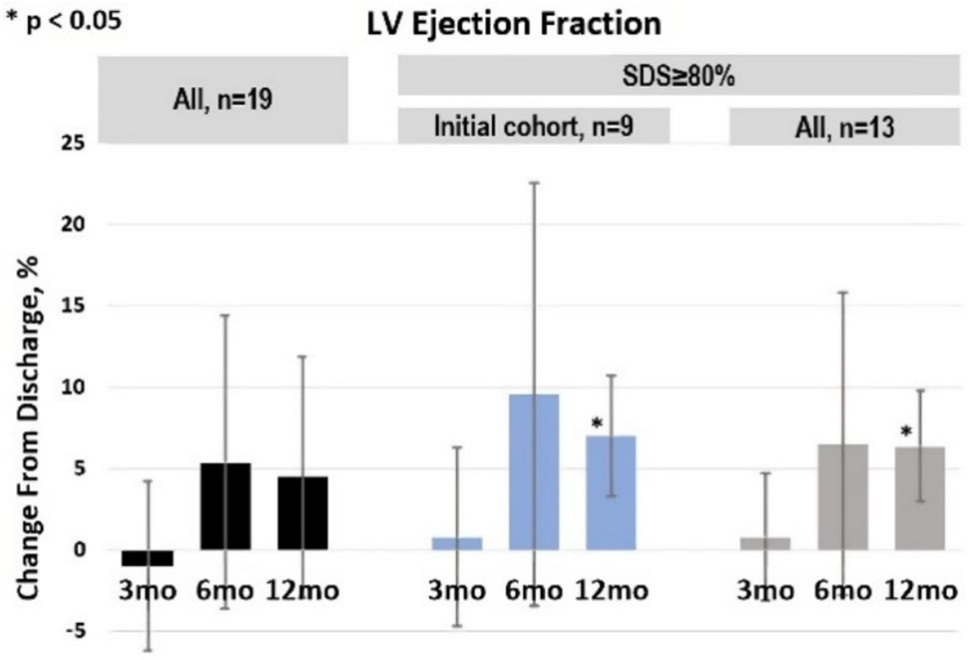
LVEF during 1-year follow-up. Total *N* = 19. Data on file at VisCardia, Inc

**Fig. 8:**
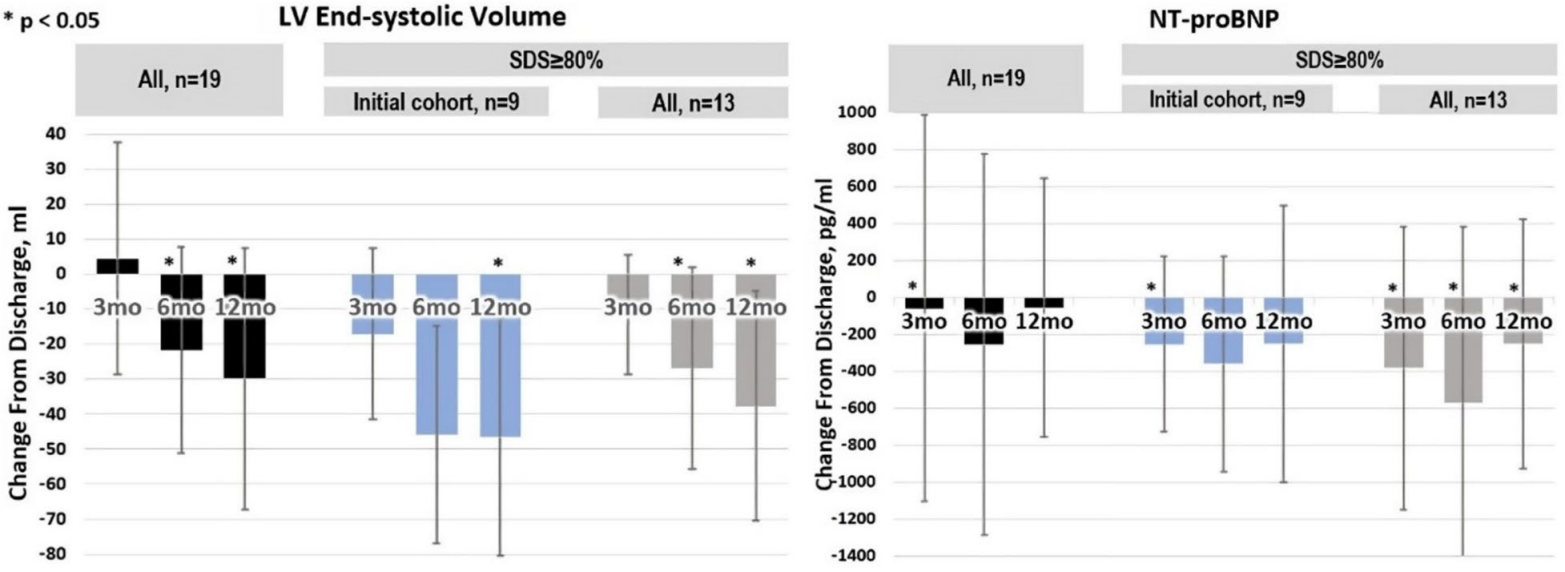
LVESV and NT-proBNP during 1-year follow-up. Total *N* = 19. Data on file at VisCardia, Inc

In the same pooled data (Figs. 7 and 8), improved cardiac performance and potential remodeling during SDS therapy were shown in several ways including increased left-ventricular ejection fraction (LVEF), reduced left-ventricular end-systolic volume (LVESV), and NT-proBNP. The reduction in LVESV over time also suggests that there may be reverse remodeling [11].

Functional capacity, activity time, and fatigue have been correlated with 1-year transplant or LVAD free survival. In a study of 345 patients, frailty was assessed as non-frail (43%), prefrail (40%) or frail (17%) and followed for 1 year for event-free survival defined as LVAD implantation, heart transplant, or death. Recently, the International Society of Heart and Lung Transplant has published a consensus statement on the assessment and management of frailty in advanced heart failure [10]. Recognizing the connection between frailty, functional capacity, and survival, the pivotal trial will evaluate functional capacity as one of the major end-points as well as capture disease progression defined as death or need for heart transplant or LVAD.

## Team approach

Recognizing heart failure as a complex syndrome creates an alliance between the HF cardiologist, other medical and surgical colleagues, as well as allied health professionals. Sokos [11] suggests that “The proposed HF-specific multidisciplinary team comprises cardiologists, surgeons, advanced practice providers, clinical pharmacists, specialty nurses, dieticians, physical therapists, psychologists, social workers, immunologists, and palliative care clinicians.” The multidisciplinary team (MDT) concept has two dimensions: vertical and horizontal. Vertically, the team requires the wholistic approach of care by the spectrum of healthcare professionals from physicians through allied health professionals. Horizontally, the MDT requires the skills of an array of physician specialties as the patients experience disease progression and cope with the many co-morbidities that accompany heart failure like diabetes, renal dysfunction, arrhythmias, and the need for advanced heart failure therapies. The heart failure specialist must collaborate with primary care, interventional cardiology, electrophysiology, nephrology, psychology, psychiatry, cardiac surgery, transplant surgery, neurology, etc. In most advanced heart failure centers, these relationships are well established but have evolved over time as new technologies and treatment approaches are developed. As Morton et al. [12] have stated, “It is clear that further breakthroughs are needed and with them the heart failure multidisciplinary teams will be paramount to deliver increasingly specialized and complex care to a growing population.” With the introduction of SDS therapy, a relatively new alliance between the HF cardiologist and the minimally invasive endoscopic surgeon will be created. The SDS implant leverages the routine of a laparoscopic procedure and adds to the portfolio of cases the surgeon can perform, while the cardiologist and cardiac anesthesiologist support the heart failure patient through surgery and recovery. By introducing outpatient surgical techniques into the clinical workflow for this patient population, the competition for high acuity beds can be eased and patients can be efficiently managed through outpatient services. Interrogating and programming the SDS device may depend on the resources and expertise of the cardiac device clinic or can be an expansion of the heart failure program. Most heart failure programs that include an LVAD program routinely interrogate and program the LVAD’s often while assessing hemodynamics or echocardiograms. Thus, device follow-up will leverage the local existing pathways of implantable heart failure treatment devices.

## Discussion

The feasibility of SDS therapy has been studied and established through a series of pre-clinical and clinical studies. The key milestone studies are listed below:

- Bauer P, et al., 2017: SDS improved hemodynamics including cardiac output in fluid overloaded porcine (*N* = 16) through modulation of ITP [13].
- Beeler R, et al., 2017: Chronic SDS sustains improved cardiac performance in patients for up to 1 year (*N* = 24) using a modified three chamber pacemaker [14].
- Jorbenadze A, et al., 2020 and 2022: First-in-human study and extension of dedicated SDS system for HFrEF demonstrated improved exercise, LVEF and QOL (*N* = 15/*N* = 4) to warrant large randomized, controlled trial [6, 7].
- Fudim M, et al., 2024: RECOVER-HF Pilot initiated to evaluate safety profile and chronic measures of heart failure in a randomized, double-blinded feasibility protocol (*N* = 35) as a precursor for a pivotal study in the USA. Enrollment is complete, 6-month primary endpoint follow-up data presented [8].

In particular, symptomatic HFrEF patients on GDMT have demonstrated that SDS therapy has beneficial effects across clinical dimensions of cardiac performance, exercise capacity, and QOL. There has also been a favorable safety profile with no device or procedural associated adverse effects in the clinical trials to date. These preliminary findings have led to the FDA granting breakthrough device designation. This designation allows for a partnership between the FDA and the investigators, facilitating a streamlined approach to the development of clinical trials to build the portfolio of evidence allowing this technology to potentially reach patients sooner. The goal is to reach patients with on-going heart failure symptoms despite maximally tolerated guideline directed medical therapy prior to the need of transplant or LVAD while improving quality of life and reducing heart failure economic burden.

As the next step, a randomized pivotal trial is being developed in the USA to test the efficacy and safety of synchronized diaphragmatic pacing. The initial trials have been promising and the pathophysiologic and biologic mechanisms are being defined. These data have informed the development of the next investigations. An application is currently under review with FDA, and a double-blinded multicenter clinical trial is anticipated to begin in 2025.

Although the target of the trial is the group of patients remaining symptomatic despite GDMT, there are recent data suggesting that there remains significant residual risk even on GDMT with improved symptoms. This was illustrated by an analysis from the EMPEROR-Reduced Trial [15], showing that although the addition of a SGLT2i reduced heart failure events compared to placebo, there is still significant ongoing risk of cardiovascular death or heart failure hospitalizations in the active treatment arm of the trial.

SDS therapy target patient population overlaps with patients who may be candidates for cardiac contractilty modulation (CCM) and baroreflex activation therapy (BAT) (Table 1). CCM delvers biphasic electric stimulation to the ventricle during the absolute refractory period and is thought to increase contractility by increasing intracellular calcium metabolism [16]. BAT stimulates the carotid baroreceptor by electrical impulses from an implanted pulse generator resulting in a centrally mediated decrease in sympathetic activity and an increase in the parasympathetic outflow [17]. Although the mechanism of SDS therapy is still being investigated, it is thought to be mediated by altering the loading conditions of the heart including reducing afterload and improving cardiac performance. Thus, each of these device therapies mitigate heart failure through very different targets. One advantage of SDS is that it is not dependent on increased adrenergic activity or the ability to electrically stimulate part of the myocardium which could be limited by significant fibrosis. Instead, SDS likely changes the performance of the heart within the circulatory system allowing for decreased work and increased cardiac output.

**Table 1:** Summary of device trials including synchronized diaphragmatic pacing, baroreflex activation therapy (BAT), and cardiac contractility management (CCM) published to date

Study/type	Sponsor/Device	Target population	*N*	Inclusion	Exclusion (most relevant)
VisONE FIM/open label/extension	VisCardia/SDS	Symptomatic HFrEF on GDMT	15/19	• NYHA class II/III • EF ≤ 35% • Sinus Rhythm • NT-proBNP>500	• QRSD ≥ 140 ms • HR > 140 bpm • Systolic BP <80 or > 170 mmHG • End-organ failure • Intravenous inotropes
RECOVER-HF Pilot/randomized, controlled, double-blinded	VisCardia/SDS	Symptomatic HFrEF on GDMT	35	• NYHA class II/III • EF ≤ 40% • QRSD ≤ 130 ms • <10% ectopy	HR > 140 bpm • Systolic BP <80 or > 170 mmHG • End-organ failure • NT-proBNP < 500 • Intravenous inotropes
BEAT-HF/randomized, controlled	CVRx/BAT	Symptomatic HFrEF on GDMT	323	• NYHA class III • EF ≤ 35% • NT-pro BNP > 400 and < 1600 pg/ml • Compatible carotid artery	• CRT indication • Autonomic neuropathy • Stage D heart failure • Bradycardia or tachycardia • Recurring hypotension • Pulmonary edema • Angina/MI/CVA/CABG/PTCA/SCD • Intravenous inotropes
FIX-HF-5 C/randomized, controlled	Impulse dynamics/CCM	Medically refractory moderate to severe heart failure	160	• EF ≥ 25 or ≤ 45 % • NYHA class III/IV • Stable medical therapy • ICD if EF < 35%	• VO2 < 9 or > 20 • Limited exercise tolerance not due to HF • Angina/ongoing ischemia • AFIB/conduction delays • Recent MI/CABG • Renal failure • >8900 ectopic beats/24 h • Intravenous inotropes

Table 1 summarizes all the trials published to date with synchronized diaphragmatic pacing (SDS) as well as baroreflex activation therapy (BAT) and cardiac contractility management (CCM) device trials for comparison.

## Limitations

As SDS is a novel technology with limited human data, clinical outcomes including impact on heart failure hospitalizations, need for LVAD or heart transplant, and survival is not yet available. In addition, there are no trials comparing SDS to approved device based heart failure treatments including CCM or BAT.

## Conclusion

SDS has shown great promise in addressing the symptomatic burden of heart failure despite maximally tolerated GDMT. This therapeutic option builds on the concepts of the heart failure team approach to include interdisciplinary specialists at the appropriate time in the patient’s disease progression to maintain their QOL and potentially provide for an improvement in cardiac function over time. SDS therapy has demonstrated feasibility in human clinical trials and is awaiting the initiation of a large multicenter US clinical trial as an FDA-designated breakthrough device. It is anticipated that success of this novel technology will lead to further evolution to deliver SDS through additional configurations such as a leadless pacing system and integration into other CIED systems.

## References

[1] Heidenreich PA, Bozkurt B, Aguilar D, Allen LA, Byun JJ, Colvin MM, Deswal A, Drazner MH, Dunlay SM, Evers LR, Fang JC, Fedson SE, Fonarow GC, Hayek SS, Hernandez AF, Khazanie P, Kittleson MM, Lee CS, Link MS, Milano CA, Nnacheta LC, Sandhu AT, Stevenson LW, Vardeny O, Vest AR, Yancy CW (2022) 2022 AHA/ACC/HFSA guideline for the management of heart failure: a report of the American College of Cardiology/American Heart Association Joint Committee on Clinical Practice Guidelines. J Am Coll Cardiol. 79(17):e263–e421. 10.1016/j.jacc.2021.12.012 35379503 10.1016/j.jacc.2021.12.012

[2] Allen LA, Stevenson LW, Grady KL, Goldstein NE, Matlock DD, Arnold RM, Cook NR, Felker GM, Francis GS, Hauptman PJ, Havranek EP, Krumholz HM, Mancini D, Riegel B, Spertus JA; American Heart Association; Council on Quality of Care and Outcomes Research; Council on Cardiovascular Nursing; Council on Clinical Cardiology; Council on Cardiovascular Radiology and Intervention; Council on Cardiovascular Surgery and Anesthesia (2012) Decision making in advanced heart failure: a scientific statement from the American Heart Association. Circulation. 125(15):1928-52. 10.1161/CIR.0b013e31824f2173 10.1161/CIR.0b013e31824f2173 PMC3893703 22392529

[3] Hawkins NM, Bennett MT, Andrade JG, Virani SA, Krahn AD, Ignaszewski A, Toma M (2015) Review of eligibility for cardiac resynchronization therapy. Am J Cardiol. 116(2):318–24. 10.1016/j.amjcard.2015.04.026 25975724 10.1016/j.amjcard.2015.04.026

[4] Salah HM, Goldberg LR, Molinger J, Felker GM, Applefeld W, Rassaf T, Tedford RJ, Mirro M, Cleland JGF, Fudim M (2022) Diaphragmatic function in cardiovascular disease JACC Review Topic of the Week. J Am Coll Cardiol. 80(17):1647–1659 36265961 10.1016/j.jacc.2022.08.760

[5] Fudim M, Mirro M, Goldberg LR (2022) Synchronized diaphragmatic stimulation for the treatment of symptomatic heart failure: a novel implantable therapy concept. JACC BTS 7(3):322–323 10.1016/j.jacbts.2022.02.012 PMC8993902 35411323

[6] Jorbenadze A, Young R, Shaburishvili T, Demyanchuk V, Buriak V, Todurov B, Rudenko K, Zuber M, Stämpfli SF, Tanner FC, Erne P, Mirro M, Fudim M, Goldberg LR, Cleland JGF (2022) Synchronized diaphragmatic stimulation for heart failure using the VisONE system: a first-in-patient study. ESC Heart Fail. 9(4):2207–2214. 10.1002/ehf2.13984 35619238 10.1002/ehf2.13984 PMC9288796

[7] Jorbenadze A, Goldberg LR, Shaburishvili T, Zuber M, Mirro M, Fudim M (2022) Synchronized diaphragmatic stimulation for heart failure with a reduced left ventricular ejection fraction using the VisONE system: a first-in-patient study with extended population. Struct Heart. 6(6):100103. 10.1016/j.shj.2022.100103 37288118 10.1016/j.shj.2022.100103 PMC10242567

[8] Fudim M, Becker G, Shaburishvili T, Chiabrishvili D, Zirakashvili T, Oniani B, Shaishmelashvili G, Mirro M, Goldberg L (2025) RECOVER-HF Pilot Study: synchronized diaphragmatic stimulation for HFrEF therapy. Presented at THT

[9] Hayes DL, Boehmer JP, Day JD, Gilliam FR 3rd, Heidenreich PA, Seth M, Jones PW, Saxon LA (2011) Cardiac resynchronization therapy and the relationship of percent biventricular pacing to symptoms and survival. Heart Rhythm. 8(9):1469–75 21699828 10.1016/j.hrthm.2011.04.015

[10] Denfeld QE, Jha SR, Fung E, Jaarsma T, Maurer MS, Reeves GR, Afilalo J, Beerli N, Bellumkonda L, De Geest S, Gorodeski EZ, Joyce E, Kobashigawa J, Mauthner O, McDonagh J, Uchmanowicz I, Dickson VV, Lindenfeld J, Macdonald P (2023) Assessing and managing frailty in advanced heart failure: an International Society for Heart and Lung Transplantation consensus statement. J Heart Lung Transplant. S1053–2498(23):02028–4. 10.1016/j.healun.2023.09.013 10.1016/j.healun.2023.09.013 38099896

[11] Sokos G, Kido K, Panjrath G et al (2023) (2023) Multidisciplinary care in heart failure services. J Card Fail 29(6):943–958. 10.1016/j.cardfail.2023.02.011 36921886 10.1016/j.cardfail.2023.02.011

[12] Sokos G, Kido K, Panjrath G, Benton , Page R, Patel J, Smith PJ, Korous S, Guglin M (2018) Multidisciplinary team approach to heart failure management. Heart 104(16), 1376

[13] Bauer P, Snell J, Wheeler T, Chinchoy E, Mirro M (2017) Animal model of diaphragmatic stimulation effects on cardiovascular function. Presented at HFSA Abstract 335

[14] Beeler R, Schoenenberger A, Zuber M, Bauer P, Erne S, Schlaepfer R, Erne P (2017) Sustained improvements to ventricular function due to asymptomatic diaphragmatic stimulation. Presented at HFSA 2017, Abstract 337

[15] Packer M, Anker SD, Butler J, Filippatos G, Pocock SJ, Carson P, Januzzi J, Verma S, Tsutsui H, Brueckmann M, Jamal W, Kimura K, Schnee J, Zeller C, Cotton D, Bocchi E, Böhm M, Choi DJ, Chopra V, Chuquiure E, Giannetti N, Janssens S, Zhang J, Gonzalez Juanatey JR, Kaul S, Brunner-La Rocca HP, Merkely B, Nicholls SJ, Perrone S, Pina I, Ponikowski P, Sattar N, Senni M, Seronde MF, Spinar J, Squire I, Taddei S, Wanner C, Zannad F. EMPEROR-Reduced Trial Investigators (2020) Cardiovascular and renal outcomes with empagliflozin in heart failure. N Engl J Med. 383(15):1413-1424. 10.1056/NEJMoa2022190 10.1056/NEJMoa2022190 32865377

[16] Pipilas DC, Hanley A, Singh JP, Mela T (2023) Cardiac contractility modulation for heart failure: current and future directions. J Soc Cardiovasc Angiogr Interv. 2(6):101176. 10.1016/j.jscai.2023.101176 39131075 10.1016/j.jscai.2023.101176 PMC11307863

[17] Hoppe UC, Brandt MC, Wachter R, Beige J, Rump LC, Kroon AA, Cates AW, Lovett EG, Haller H (2012) Minimally invasive system for baroreflex activation therapy chronically lowers blood pressure with pacemaker-like safety profile: results from the Barostim neo trial. J Am Soc Hypertens. 6(4):270–6. 10.1016/j.jash.2012.04.004 22694986 10.1016/j.jash.2012.04.004

